# Supplementary material for: Deficiency in PHD2-mediated hydroxylation of HIF2α underlies Pacak-Zhuang syndrome
Source: Commun Biol. 2024 Feb 28;7:240. doi: 10.1038/s42003-024-05904-4 (PMC10902354; doi:10.1038/s42003-024-05904-4)
Supplement: Supplementary file 2 — Supplementary Information [file 42003_2024_5904_MOESM2_ESM.pdf]

Deficiency in PHD2-mediated hydroxylation of HIF2α underlies Pacak-Zhuang syndrome

Ferens et al.

Supplementary Information

Supplementary Table 1. HIF1α and HIF2α peptides used in this study

Non-disease associated HIFα-CODD peptides	Sequence	
WT HIF2α	ELDLETLAPYIPMDGEDFQL	
WT-OH HIF2α	ELDLETLA[Hyp]YIPMDGEDFQL	
WT HIF1α	DLDLEMLAPYIPMDDDFQL	
T528M HIF2α	ELDLEMLAPYIPMDGEDFQL	
ΔG537 HIF2α	ELDLETLAPYIPMDEDFQL	
**Pacak-Zhuang syndrome mutant peptides**	**Sequence**	**Pacak-Zhuang syndrome (Subclass)**
L529P	ELDLET**P**APYIPMDGEDFQL	1 (a)
A530T	ELDLETL**T**PYIPMDGEDFQL	1 (a, b)
A530V	ELDLETL**V**PYIPMDGEDFQL	1 (a, b, c)
A530E	ELDLETL**E**PYIPMDGEDFQL	1 (b, c)
P531A	ELDLETLA**A**YIPMDGEDFQL	1 (c)
P531S	ELDLETLA**S**YIPMDGEDFQL	1 (b, c)
P531L	ELDLETLA**L**YIPMDGEDFQL	1 (b, c)
Y532C	ELDLETLAP**C**IPMDGEDFQL	1 (a, c)
D539N	ELDLETLAPYIPMDGE**N**FQL	1 (a, c)
D539Y	ELDLETLAPYIPMDGE**Y**FQL	1 (c)
L542P	ELDLETLAPYIPMDGEDFQ**P**	1 (b)
Y532H	ELDLETLAP**H**IPMDGEDFQL	2
I533V	ELDLETLAPY**V**PMDGEDFQL	2
P534L	ELDLETLAPYI**L**MDGEDFQL	2
P534R	ELDLETLAPYI**R**MDGEDFQL	2
M535I	ELDLETLAPYIP**I**DGEDFQL	2
M535T	ELDLETLAPYIP**T**DGEDFQL	2
G537W	ELDLETLAPYIPMD**W**EDFQL	2
G537R	ELDLETLAPYIPMD**R**EDFQL	2
D539E	ELDLETLAPYIPMDGE**E**FQL	2
F540L	ELDLETLAPYIPMDGED**L**QL	2

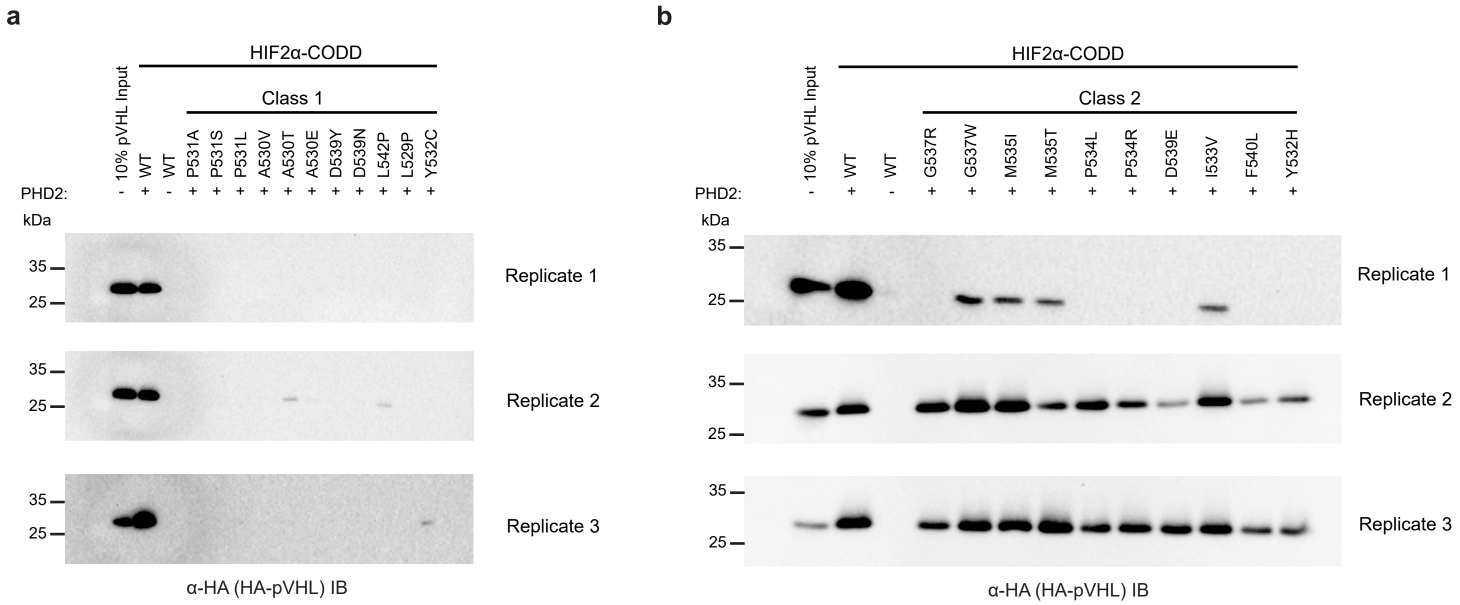

Supplementary Figure 1. Pacak-Zhuang syndrome Class 1 and Class 2 mutants are not reliably distinguished by the indirect hydroxylation assay. Immobilized HIF2α-CODD peptides were hydroxylated by PHD2. HA-pVHL was pulled down by hydroxylated peptides and an α-HA-pVHL Western blot was used to assess the extent of hydroxylation (a) Three replicates of the indirect hydroxylation assay of Class 1 mutants. (b) Three replicates of the indirect hydroxylation assay of Class 2 mutants.

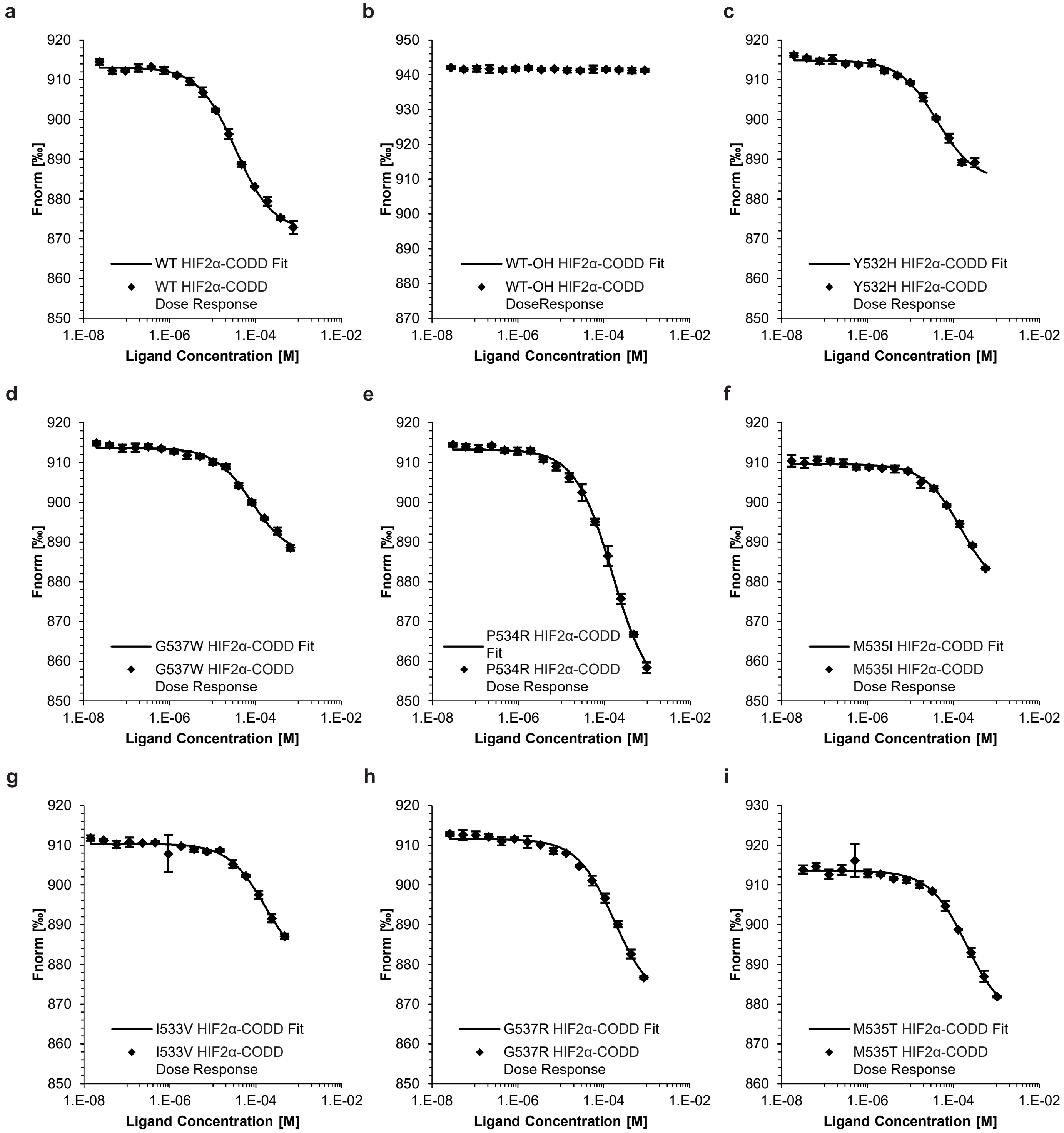

Supplementary Figure 2. (Continues on next page)

j

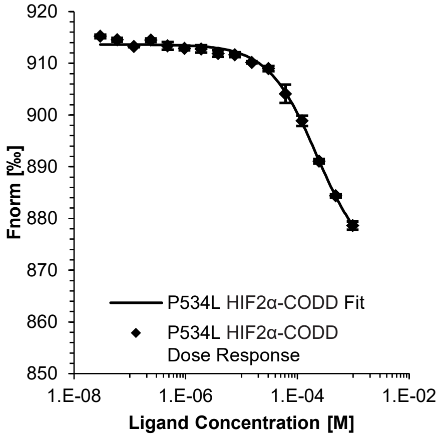

k

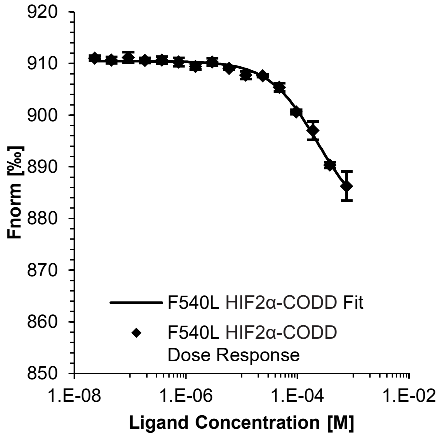

l

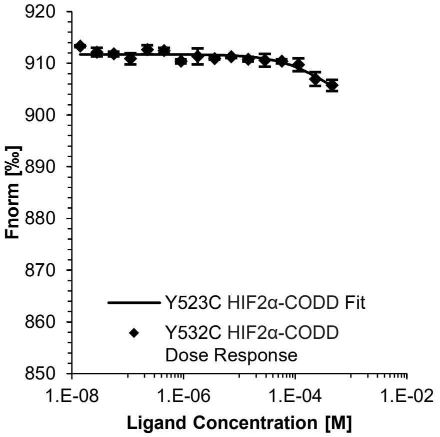

m

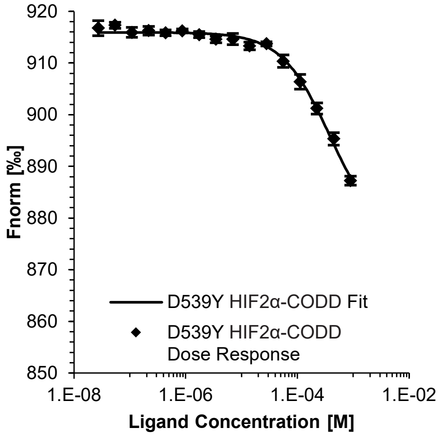

n

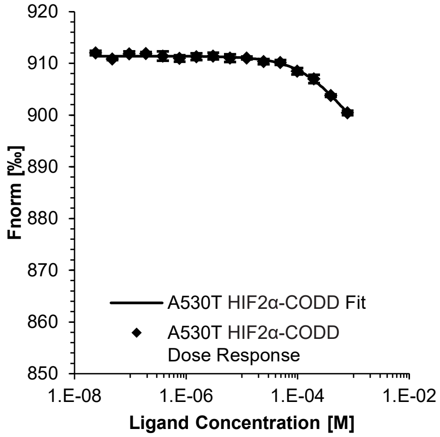

o

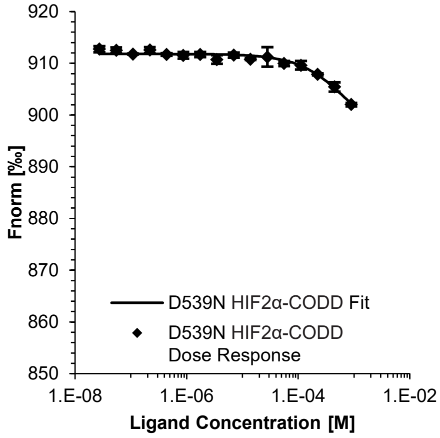

p

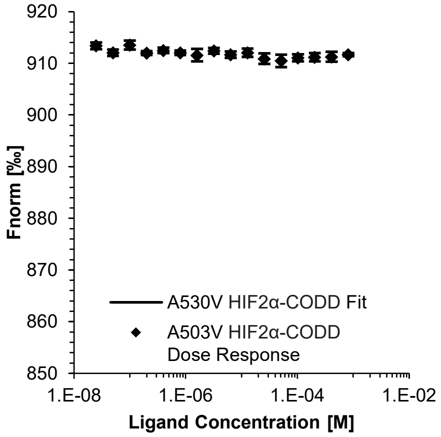

q

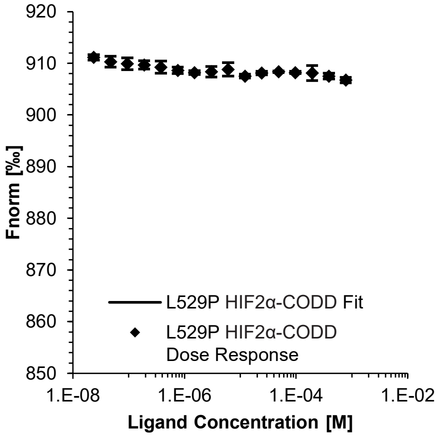

r

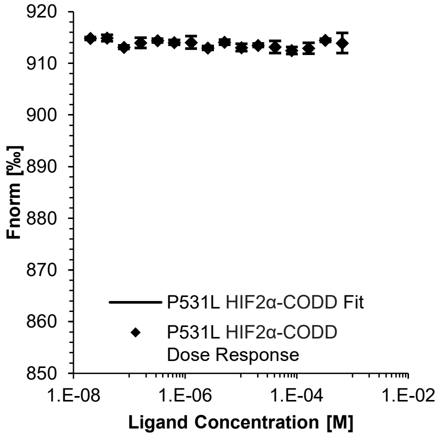

Supplementary Figure 2 continued. **(continues on next page)**

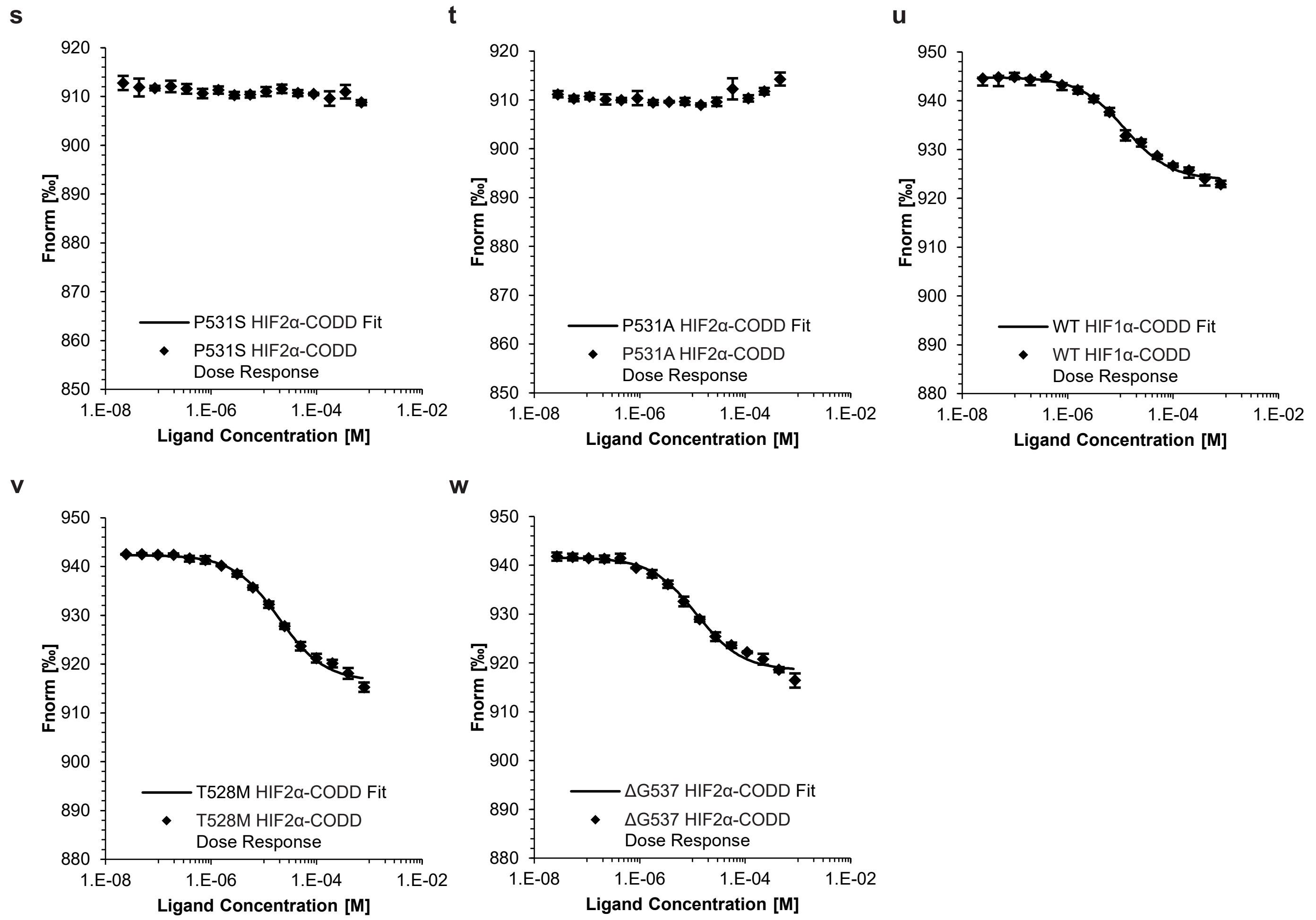

Supplementary Figure 2 continued. Individual (non-normalized) PHD2 MST binding curves for each HIFα-CODD peptide examined in this study in the order in which they appear in the main figures: (a) WT HIF2α-CODD, (b) WT-OH HIF2α-CODD, (c) Y532H HIF2α-CODD, (d) G537W HIF2α-CODD, (e) P534R HIF2α-CODD, (f) M535I HIF2α-CODD, (g) I533V HIF2α-CODD, (h) G537R HIF2α-CODD, (i) M535T HIF2α-CODD, (j) P534L HIF2α-CODD, (k)F540L HIF2α-CODD, (l) Y532C HIF2α-CODD, (m) D539Y HIF2α-CODD, (n) A530T HIF2α-CODD, (o) D539N HIF2α-CODD, (p) A530V HIF2α-CODD, (q) L529P HIF2α-CODD, (r) P531L HIF2α-CODD, (s) P531S HIF2α-CODD, (t) P531A HIF2α-CODD, (u) WT HIF1α-CODD, (v) T528M HIF2α-CODD, (w) ΔG537 HIF2α-CODD.

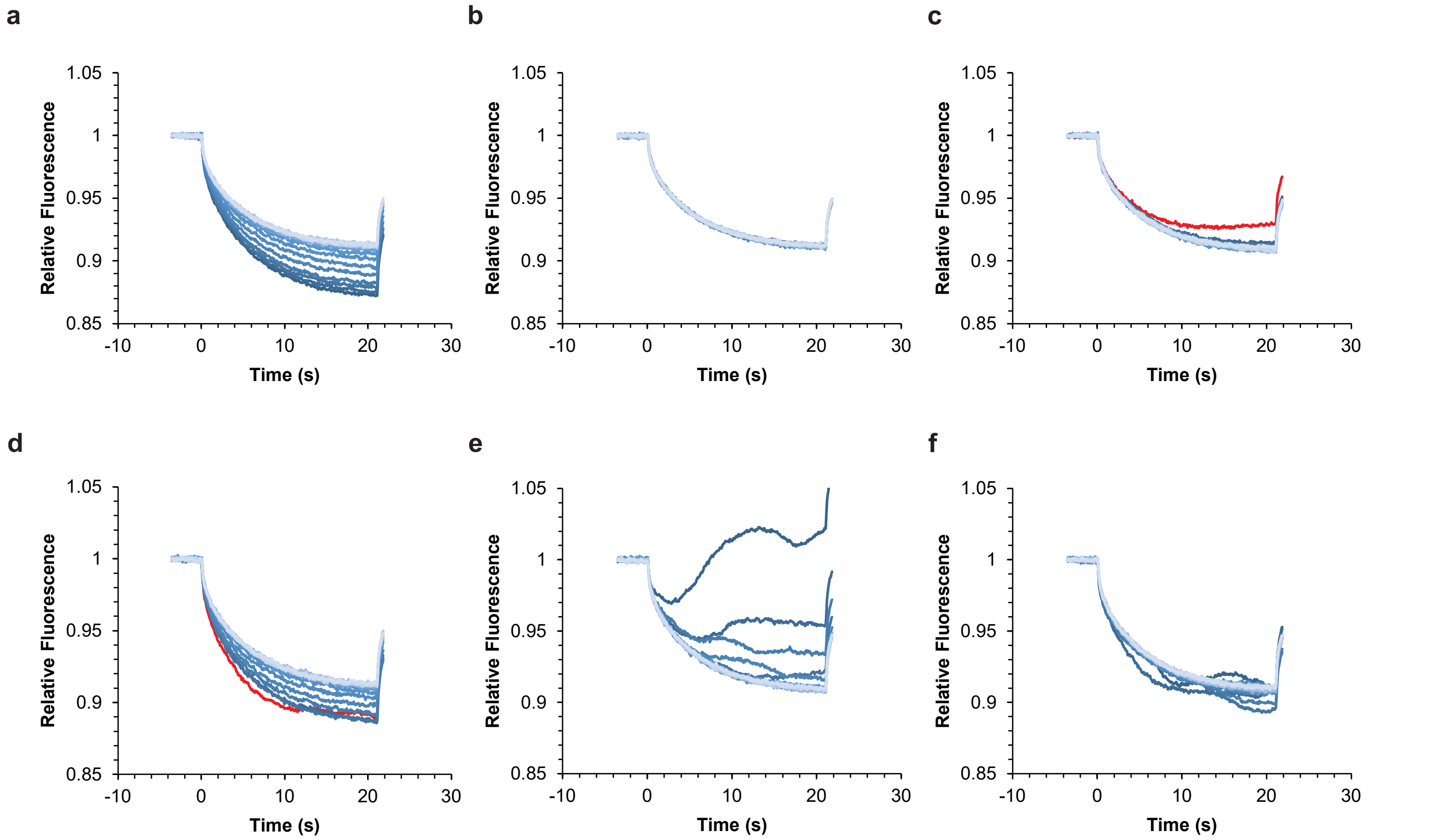

Supplementary Figure 3. Some HIF2α-CODD mutants cause aggregation of PHD2 in MST experiments. Representative MST time traces of PHD2 with (a) WT HIF2α-CODD showing binding and no aggregation. (b) WT-OH HIF2α-CODD showing no binding and no aggregation, (c) P531A HIF2α-CODD showing some evidence of aggregation at the highest concentration examined, (d) Y532H HIF2α-CODD showing evidence of aggregation at the highest concentration examined, (e) L542P HIF2α-CODD showing severe aggregation at multiple concentrations and (f) D539E HIF2α-CODD showing severe aggregation at multiple concentrations. All curves are coloured light blue to dark blue corresponding to lowest to highest concentration of HIF2α-CODD. Curves colored red in panels c and d represent the highest concentrations of HIF2α-CODD in these experiments which were omitted from our final analysis due to observed aggregation of PHD2 at these high concentrations of peptide.

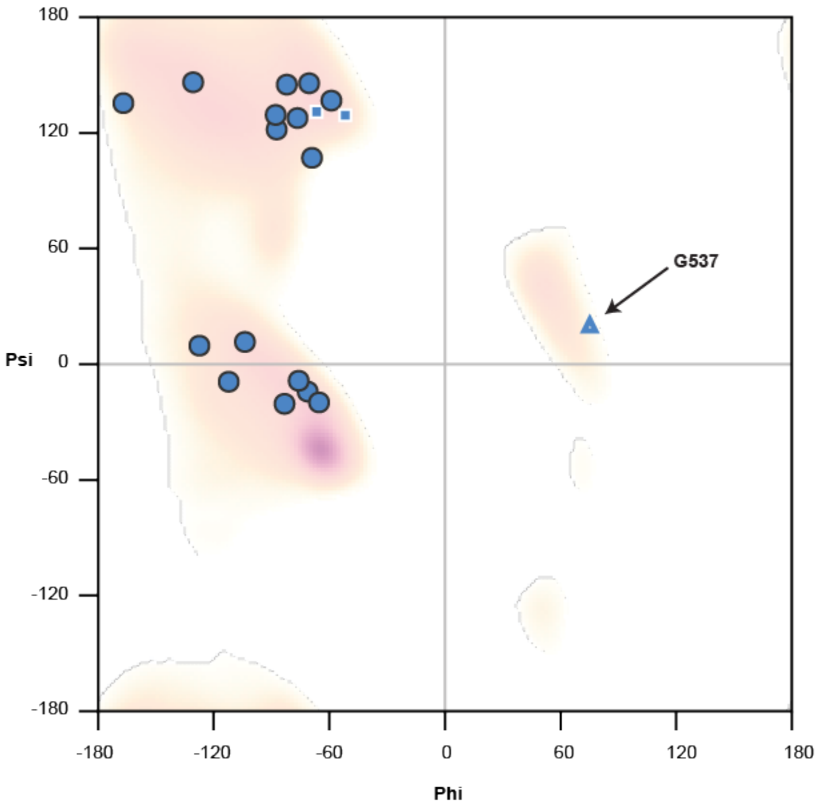

Supplementary Figure 4. Ramachandran plot of the HIF2α-CODD peptide from our PHD2/HIF2α-CODD complex. Proline residues are plotted as squares, glycine residues are plotted as triangles and all other amino acids are plotted as circles. The location of G537 on the plot is indicated. The favored (purple) and allowed regions (yellow) for most amino acids excluding glycine and proline are displayed. G537 occupies a position near the edge of the allowed region for most other amino acids.

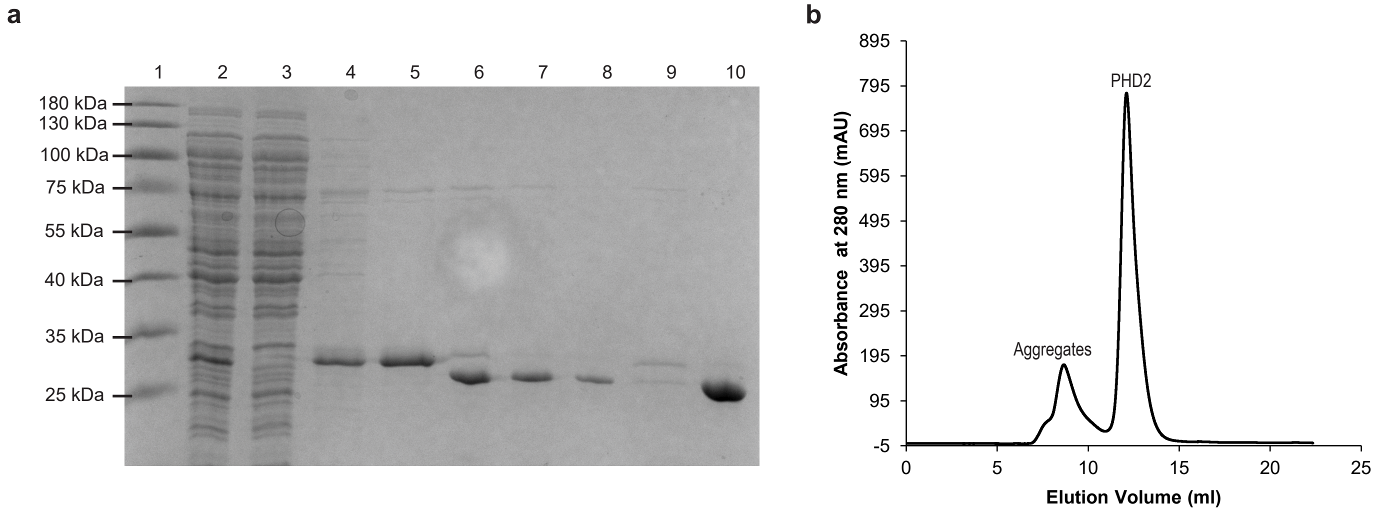

S**upplementary Figure 5.** Purification of PHD2. His_6-PHD2 (residues 181-426) was purified from BL21(DE3) E .coli cells using Ni-NTA agarose beads and size exclusion chromatography (a) Coomassie stained SDS-PAGE of PHD2 purification. Lanes: 1) Protein ladder, 2) BL21(DE3) cell lysate, 3) Ni-NTA column flow through, 4) Ni-NTA column wash, 5) PHD2 elution from NI-NTA column, 6) Thrombin digestion to remove His_6-tag, 7) Reverse purification flow through, 8) Reverse purification 5 mM Imidazole wash, 9) Material remaining on column after reverse purification, 10) Pooled PHD2 containing Superdex 75 increase fractions. (b) Superdex 75 increase chromatogram of PHD2 purification, the peak containing aggregatted material and PHD2 are indicated.

Figure 1a

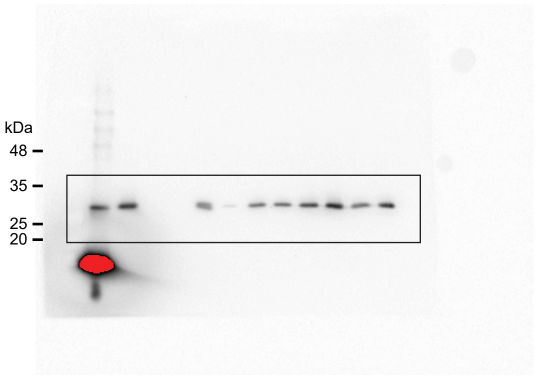

Supplementary Figure 1a

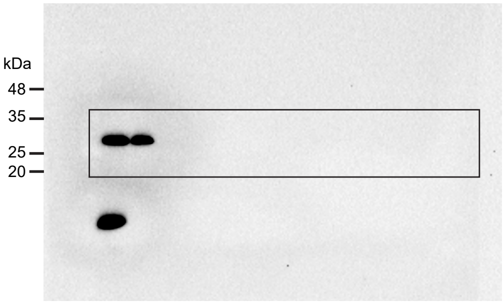

Replicate 1

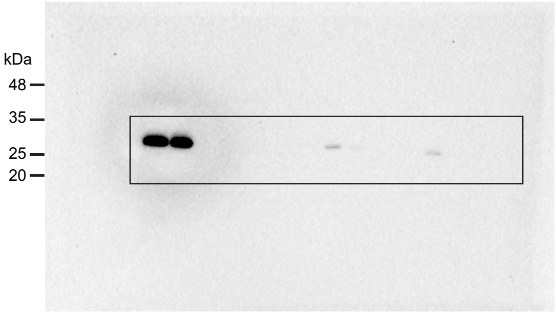

Replicate 2

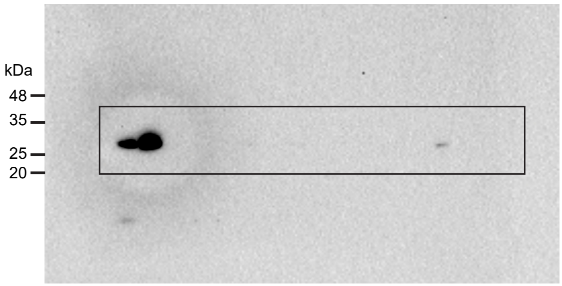

Replicate 3

Supplementary Figure 1b

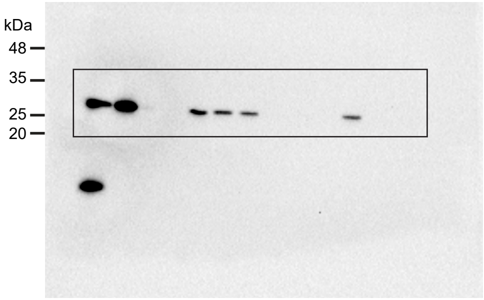

Replicate 1

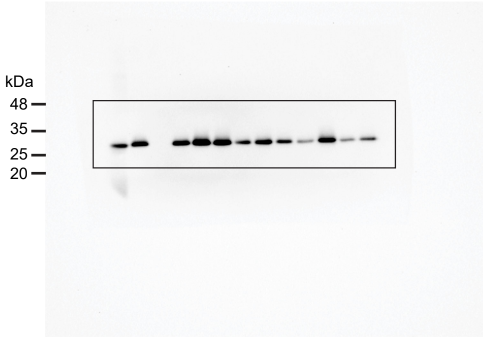

Replicate 2

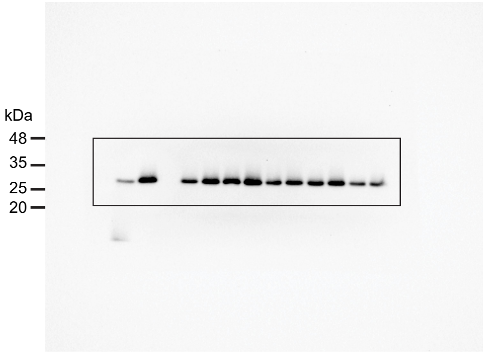

Replicate 3

Supplementary Figure 6. Uncropped western blots. The approximate area used in the indicated figures is shown as a black box. All blots used anti-HA to detect HA-VHL pulled down in the hydroxylation assays.
