# Supplementary material for: Deficiency in PHD2-mediated hydroxylation of HIF2α underlies Pacak-Zhuang syndrome
Source: Commun Biol. 2024 Feb 28;7:240. doi: 10.1038/s42003-024-05904-4 (PMC10902354; doi:10.1038/s42003-024-05904-4)
Supplement: Supplementary file 5 — Reporting Summary [file 42003_2024_5904_MOESM5_ESM.pdf]

nature portfolio

Corresponding author(s): Michael Ohh

Last updated by author(s): Jan 11, 2024

Reporting Summary

Nature Portfolio wishes to improve the reproducibility of the work that we publish. This form provides structure for consistency and transparency in reporting. For further information on Nature Portfolio policies, see our Editorial Policies and the Editorial Policy Checklist.

Statistics

For all statistical analyses, confirm that the following items are present in the figure legend, table legend, main text, or Methods section.

n/a	Confirmed	
☐	☒	The exact sample size (n) for each experimental group/condition, given as a discrete number and unit of measurement
☐	☒	A statement on whether measurements were taken from distinct samples or whether the same sample was measured repeatedly
☐	☒	The statistical test(s) used AND whether they are one- or two-sided<br>*Only common tests should be described solely by name; describe more complex techniques in the Methods section.*
☒	☐	A description of all covariates tested
☐	☒	A description of any assumptions or corrections, such as tests of normality and adjustment for multiple comparisons
☐	☒	A full description of the statistical parameters including central tendency (e.g. means) or other basic estimates (e.g. regression coefficient) AND variation (e.g. standard deviation) or associated estimates of uncertainty (e.g. confidence intervals)
☒	☐	For null hypothesis testing, the test statistic (e.g. F, t, r) with confidence intervals, effect sizes, degrees of freedom and P value noted<br>*Give P values as exact values whenever suitable.*
☒	☐	For Bayesian analysis, information on the choice of priors and Markov chain Monte Carlo settings
☒	☐	For hierarchical and complex designs, identification of the appropriate level for tests and full reporting of outcomes
☒	☐	Estimates of effect sizes (e.g. Cohen's d, Pearson's r), indicating how they were calculated

Our web collection on statistics for biologists contains articles on many of the points above.

Software and code

Policy information about availability of computer code

Data collection	M.O. Control (Nanotemper Technologies) was used to collect MST Data
Data analysis	M.O. Affinity Analysis (Nanotemper technologies) was used for MST analysis. CCP4 software package (DIALS, PHASER, BUCCANEER, REFMAC5 and MOLPROBITY programs within CCP4) was used for x-ray diffraction processing and structure solution and validation. Coot was used for manual atomic model building. PISA (CCP4) was used to analyze protein-protein interactions in the crystal structure. Phenix was used for composite omit electron density map calculation and to extract and compile the crystallography and structure solution statistics presented in Table 3. See text for software references.

For manuscripts utilizing custom algorithms or software that are central to the research but not yet described in published literature, software must be made available to editors and reviewers. We strongly encourage code deposition in a community repository (e.g. GitHub). See the Nature Portfolio guidelines for submitting code & software for further information.

Data

Policy information about availability of data

All manuscripts must include a data availability statement. This statement should provide the following information, where applicable:

- Accession codes, unique identifiers, or web links for publicly available datasets
- A description of any restrictions on data availability
- For clinical datasets or third party data, please ensure that the statement adheres to our policy

The PHD2/HIF2α-CODD crystal structure has been deposited in the Protein Data Bank (PDB entry: 7UJV). Numerical data used to create the plots in this work is published alongside the online version of this manuscript as a supplemental file. Uncropped blot images are provided in Supplementary Fig. 6. All other data are available from the corresponding author on reasonable request.

Research involving human participants, their data, or biological material

Policy information about studies with human participants or human data. See also policy information about sex, gender (identity/presentation), and sexual orientation and race, ethnicity and racism.

Reporting on sex and gender	N/A
Reporting on race, ethnicity, or other socially relevant groupings	N/A
Population characteristics	N/A
Recruitment	N/A
Ethics oversight	N/A

Note that full information on the approval of the study protocol must also be provided in the manuscript.

Field-specific reporting

Please select the one below that is the best fit for your research. If you are not sure, read the appropriate sections before making your selection.

☒ Life sciences ☐ Behavioural & social sciences ☐ Ecological, evolutionary & environmental sciences

For a reference copy of the document with all sections, see nature.com/documents/nr-reporting-summary-flat.pdf

Life sciences study design

All studies must disclose on these points even when the disclosure is negative.

Sample size	N/A
Data exclusions	1 MST trace was removed from each the P531A and Y532H MST data sets prior to Kd determination due to signs of protein aggregation in the samples, see Supplementary Figure 3c and 3d. No Kd determination was performed on MST data for L542P and D539E mutant peptides due to detected protein aggregation in all MST traces, see Supplementary Figure 3e and 3f.
Replication	For the PHD2/HIF2a-CODD structure solution all data is derived from a single crystal. All other experiments were performed at least 3 times. For western blots, representative blots are presented in the main text and replicates are included in Supplementary Figure 1. For experiments with quantitative results, averages of all replicates are presented along with standard error and dot plot of individual data points.
Randomization	N/A
Blinding	N/A

Reporting for specific materials, systems and methods

We require information from authors about some types of materials, experimental systems and methods used in many studies. Here, indicate whether each material, system or method listed is relevant to your study. If you are not sure if a list item applies to your research, read the appropriate section before selecting a response.

Materials & experimental systems

n/a	Involved in the study	
☐	☒	Antibodies
☐	☒	Eukaryotic cell lines
☒	☐	Palaeontology and archaeology
☒	☐	Animals and other organisms
☒	☐	Clinical data
☒	☐	Dual use research of concern
☒	☐	Plants

Methods

n/a	Involved in the study	
☒	☐	ChIP-seq
☒	☐	Flow cytometry
☒	☐	MRI-based neuroimaging

Antibodies

Antibodies used	anti-HA (Cell Signaling, C29F4)
Validation	The anti-HA antibody is validated by the manufacturer via heterologous expression (https://www.cellsignal.com/products/primary-antibodies/ha-tag-c29f4-rabbit-mab/3724)

Eukaryotic cell lines

Policy information about cell lines and Sex and Gender in Research

Cell line source(s)	HEK293A human embryonic kidney epithelial cell line was obtained from ATCC.
Authentication	The HEK293A cell line was not authenticated.
Mycoplasma contamination	The HEK293A cells tested negative for mycoplasma contamination
Commonly misidentified lines (See ICLAC register)	*Name any commonly misidentified cell lines used in the study and provide a rationale for their use.*

Plants

Seed stocks	N/A
Novel plant genotypes	N/A
Authentication	N/A
